# *IDH* mutation-specific radiomic signature in lower-grade gliomas

**DOI:** 10.18632/aging.101769

**Published:** 2019-01-29

**Authors:** Xing Liu, Yiming Li, Shaowu Li, Xing Fan, Zhiyan Sun, Zhengyi Yang, Kai Wang, Zhong Zhang, Tao Jiang, Yong Liu, Lei Wang, Yinyan Wang

**Affiliations:** ^1^Beijing Neurosurgical Institute, Capital Medical University, Beijing, China; ^2^Neurological Imaging Center, Beijing Neurosurgical Institute, Capital Medical University, Beijing, China; ^3^Department of Neurosurgery, Beijing Tiantan Hospital, Capital Medical University, Beijing, China; ^4^Brainnetome Center, Institute of Automation, Chinese Academy of Sciences, Beijing, China; ^5^Department of Nuclear Medicine, Beijing Tiantan Hospital, Capital Medical University, Beijing, China; ^6^Center of Brain Tumor, Beijing Institute for Brain Disorders, Beijing, China; ^7^China National Clinical Research Center for Neurological Diseases, Beijing, China; ^8^Chinese Glioma Genome Atlas Network (CGGA) and Asian Glioma Genome Atlas Network (AGGA); ^9^National Laboratory of Pattern Recognition, Institute of Automation, Chinese Academy of Sciences, Beijing, China; ^*^Equal contribution

**Keywords:** isocitrate dehydrogenase, lower grade gliomas, radiomic signature, transcriptome-radiomic analysis, prognostic signature

## Abstract

Unravelling the heterogeneity is the central challenge for glioma precession oncology. In this study, we extracted quantitative image features from T2-weighted MR images and revealed that the *isocitrate dehydrogenase* (*IDH*) wild type and mutant lower grade gliomas (LGGs) differed in their expression of 146 radiomic descriptors. The logistic regression model algorithm further reduced these to 86 features. The classification model could discriminate the two types in both the training and validation sets with area under the curve values of 1.0000 and 0.9932, respectively. The transcriptome-radiomic analysis revealed that these features were associated with the immune response, biological adhesion, and several malignant behaviors, all of which are consistent with biological processes that are differentially expressed in *IDH* wild type and *IDH* mutant LGGs. Finally, a prognostic signature showed an ability to stratify IDH mutant LGGs into high and low risk groups with distinctive outcomes. By extracting a large number of radiomic features, we identified an *IDH* mutation-specific radiomic signature with prognostic implications. This radiomic signature may provide a way to non-invasively discriminate lower-grade gliomas as with or without the IDH mutation.

## INTRODUCTION

Diffuse gliomas, graded from II to IV according to the World Health Organization (WHO) criteria, are the most common and lethal primary tumors of the central nervous system. Lower grade gliomas (LGGs), designated as astrocytomas, oligodendrogliomas, and mixed oligoastrocytomas of grade II and III gliomas, account for approximately 43.2% of all gliomas diagnosed in adults [1–3]. Although LGGs have a relatively better therapeutic response and longer overall survival (OS) than fully malignant glioblastomas (GBM, WHO grade IV), they eventually transform to higher grade tumors with greater mortality [4, 5].

Isocitrate dehydrogenase (*IDH*) enzymes are crucial for the tricarboxylic acid cycle, catalyzing the oxidative decarboxylation of isocitrate. Mutations of *IDH* genes result in production of the oncometabolite 2-hydroxyglutarate (2-HG) instead of α-ketoglutarate [6]. Previous research studies found that *IDH* mutations are a causative event in gliomagenesis, as well as a diagnostic, classification, and prognostic biomarker for LGG patients [7–9]. Patients harboring these mutations generally have a favorable prognosis, independent of their WHO grade [10, 11]. In light of the crucial role of *IDH* mutations in glioma management, *IDH* examination has become a routine diagnostic modality in many neuropathology centers [1, 3]. Currently, immunohistochemistry staining for *IDH1*^R132H^ using formalin-fixed, paraffin-embedded specimens is the most common approach [12–14]. Alternatively, Sanger sequencing and pyrosequencing analyses are also commonly used, especially for those suspected of harboring mutations other than *IDH1*^R132H^ [14, 15]. However, the classical determination of *IDH* status requires surgically removal of tumor tissues. A noninvasive method would be more helpful in the treatment plan and for the prognostic prediction of glioma management.

Previous studies have reported associations between imaging manifestations and *IDH* mutations. *IDH* mutant low-grade gliomas occur most frequently in the frontal lobe [11], especially in the area surrounding the rostral extension of the lateral ventricles [16]. *IDH* wild type gliomas exhibit more post-contrast enhancement on MR images than their mutant counterparts [17, 18]. Diffusion (the apparent diffusion coefficient and fractional anisotropy) and perfusion (the relative cerebral blood volume and normalized cerebral blood volume) MR imaging can also be used in distinguishing *IDH* wild type and mutant gliomas [19–21]. Importantly, recent studies showed that the oncometabolite 2-HG can be detected *in vivo* using magnetic resonance spectroscopy (MRS), providing a better option for *IDH* testing [22–24]. However, the detection of 2-HG requires a unique MRS sequence device and cannot therefore be feasibly applied in a standard clinical setting [25]. Notably, few of the above approaches are either diagnostic or quantitative.

Radiomics is a quantifying innovation that extracts large numbers of features from radiographic images using automatic data-characterization algorithms [26, 27]. In pioneering work, investigators have applied quantitative radiomics analysis to computer tomography [28], MR [29], and positron emission tomography imaging data [30], deciphering tumor phenotypes of non-small cell lung carcinoma [28], head and neck cancers [31], and breast cancers [32]. Gevaert et al. utilized shape, texture, and edge sharpness to divide GBM patients into three clusters with corresponding molecular alterations [29]. These studies highlight the potential of radiomics for quantifying and monitoring tumor-phenotypic characteristics in clinical practice [33]. In the present study, we assessed a total of 431 radiomic features, including first order statistics, shape and size based features, textural features, and wavelet features, from T2-weighted MR images. By comparing radiological and transcriptomic profiles of *IDH* mutant (*IDH*^MUT^) and *IDH* wild type (*IDH*^WT^) LGG patients, diagnostic radiomic features for the *IDH* mutations were identified and independently validated. Furthermore, transcriptomic differences between the two groups and the biological processes underlying several significant radiomic features were explored. Our results suggested that the radiomic signature can separate the *IDH*^MUT^ and *IDH*^WT^ phenotypes of LGG patients and can potentially enable the distinction between molecular subtypes of LGGs and facilitate the design of new treatments.

## RESULTS

### Demographic and clinical characteristics

A total of 158 patients diagnosed with LGG were enrolled as the training data set. Of these, 118 (74.7%) had *IDH* mutant (*IDH*^MUT^) tumors and 40 (25.3%) had *IDH* wild type (*IDH*^WT^) tumors. No significant differences were observed with respect to age, sex, WHO grade, and tumor location between the two groups. The *IDH* mutation rate in the validation data set was 75.5% (77 out of 102). The clinical and pathological characteristics of the training and the validation data sets are listed in Table 1.

**Table 1:** Clinical characteristics of Lower Grade Glioma patients in training and validation set

	Training Set			Validation Set		
	IDH^WT^ (n=40)	IDH^MUT^ (n=118)	*P*	IDH^WT^ (n=25)	IDH^MUT^ (n=77)	*P*
Age median (range), years	37 (18-61)	38 (18-63)	0.403^a^	44 (22-70)	42 (22-68)	0.754^a^
Sex						
Male	26	74	0.795^ b^	15	46	0.999^b^
Female	14	44		10	31	
WHO Grade						
II	25	89	0.115^ b^	13	62	0.005^ b^
III	15	29		12	15	
Lesion Location						
Left	24	66	0.132^ c^	10	38	0.565^ c^
Right	16	41		12	34	
Left+Right	0	11		3	5	

^a^Student’s t test; ^b^Chi-square test; ^c^Fisher’s exact test; WT = Wild Type; MUT = Mutation.

### Identification of LGGs with similar radiomic patterns

**Figure 1:**
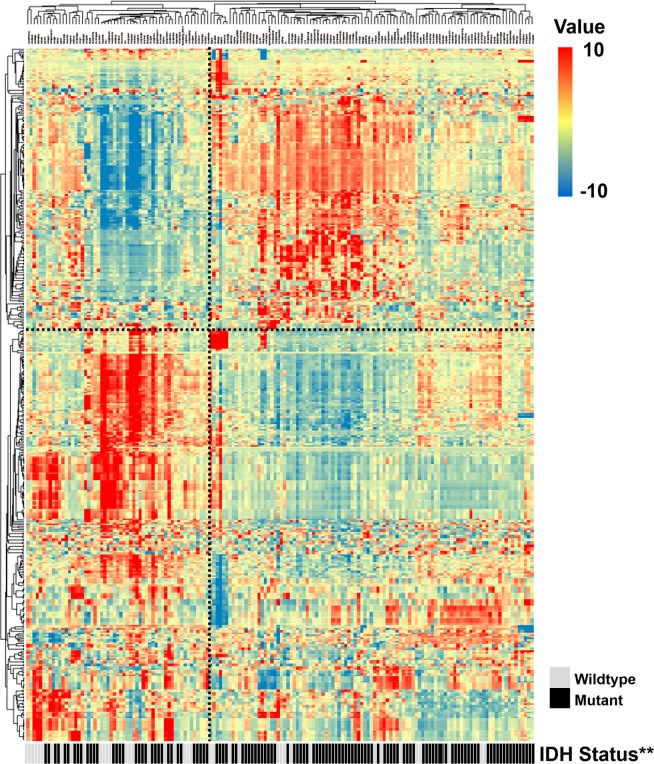
**Radiomic patterns of 431 features in LGGs.** Each column corresponds to one patient in the training cohort, and each row corresponds to one z-score-normalized radiomic feature. Unsupervised clustering between radiomic features and LGG samples revealed two distinct radiomic patterns. The second cluster showed a higher frequency of the IDH mutation (**, *P* < 0.01).

To assess the radiomic expression patterns, the quantitative radiomic features were extracted from the LGG patients in the training set. An unsupervised hierarchical clustering method with average linkage revealed two clusters of patients with similar radiomic expression patterns (Figure 1). By comparing the clinical parameters of the two clusters, we found that the second cluster was significantly associated with a high frequency of the *IDH* mutation (*P* = 0.0020, Fisher’s exact test, Figure 1), which indicates a tight association between *IDH* mutation status and quantitative radiomic features.

### Identification of the IDH-mutation specific radiomic signature

**Figure 2:**
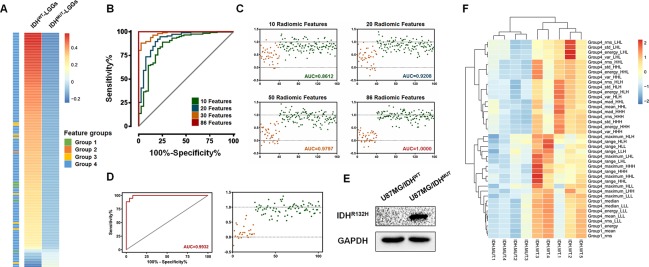
**Identification and validation of the *IDH* mutation-specific radiomic signature using the logistic regression.** (**A**) A total of 146 radiomic features were selected using SAM methods. The mean value and the corresponding groups of the differentially expressed features are listed. (**B** and **C**) In the training set, the logistic regression-derived radiomic features was able to separate LGGs into two groups with high sensitivity and specificity. The AUCs were 0.86, 0.92, 0.98 and 1.00 for 10, 20, 50 and 86 radiomic features, respectively. (**D**) Importantly, these 86 features comprised a signature enabling the distinction of LGGs into *IDH*^MUT^ and *IDH*^WT^ groups with an AUC of 0.9932. (**E**) A Western Blot assay confirmed the expression of the mutant *IDH1* protein (*IDH1*^R132H^, 1:200, DIA-H05, Dianova). (**F**) The radiogenomic analysis of xenograft gliomas of nude mice. Differential radiomic features between LGGs patients could be used to distinguish the *IDH* mutation phenotype in the xenograft model as well.

Based on previous observations, our goal was to identify a set of radiomic features that would enable the pre diction of the *IDH* mutation status in LGGs. We first screened the differences in the radiomic features between the *IDH*^WT^ and *IDH*^MUT^ LGGs using the SAM algorithm. A total of 146 features, including 5 first order statistics features (energy, entropy, mean, median and root mean square), 1 shape and size based feature (surface to volume ratio), 8 textural features (GLCM: contrast, dissimilarity, energy, entropy, difference entropy, informational measure of correlation 1; GLRLM: short run emphasis, run percentage) and 132 wavelet features (Figure 2A), were found to be expressed differentially. All the 146 screened features were listed in Supplementary Table 1.

Then, we utilized the logistic regression algorithm to select the *IDH* mutation-specific signature. A series of ROC curves with relevant AUC was delineated using the given parameters. The results showed that only ten radiomic features were needed to divide 158 LGGs into an *IDH*^WT^ group and an *IDH*^MUT^ group with an AUC of 0.86, while the AUC was 0.92 for 20 features and 0.98 for 50 features (Figure 2B and 2C). Notably, using a classification model of 86 radiomic features, the enrolled patients were correctly classified into the *IDH*^WT^ and the *IDH*^MUT^ groups (AUC = 1.00, Figure 2B and 2C).

To further validate the classification of this radiomic signature, T2-weighted images from 102 patients and *in vivo* xenograft glioma models were subjected to feature extraction and logistic regression. Consistently, the results suggested that this signature, which was comprised of 86 radiomic features, could separate the 102 LGG patients into two groups with high sensitivity and specificity (AUC=0.99, Figure 2D). Fifty-four differentially expressed radiomic features were found in xenograft model, and 40 of them (such as Energy, Mean, Median and Root mean square) were shared between patients and xenograft model (Supplementary Table 2). Moreover, by using 40 of the differential features, we could divide the experimental mice into two groups in accordance with their IDH phenotype (Figure 2E and 2F). These *in vivo* experiments further indicated the robustness of radiomic features in differentiating *IDH* mutation status. Specifically, the immune associated features were also listed in Supplementary Table 2.

### The potential molecular mechanism of IDH-mutation-specific radiomic features

CGGA and TCGA mRNA sequencing data were utilized to identify the biological processes and signaling pathways that differed significantly between the IDH^MUT^ and IDH^WT^ LGGs. Separate gene annotations of the CGGA data and the TCGA data consistently indicated that they primarily involved these biological processes: the immune response, cell adhesion, and vascular development (Supplementary Figure 1 and Supplementary Figure 2). In addition, the TCGA data suggested that the cell cycle phase and cell proliferation were also involved in the radiological manifestation of the IDH^WT^ LGGs (Supplementary Figure 2).

Meanwhile, to attempt to determine the exact biological significance of each radiomic feature, 48 LGGs samples with both transcriptomic and radiomics data were subjected to correlation analysis and gene annotation. The results indicated that the *IDH*-specific radiomic features, such as the surface to volume ratio (SVR) and entropy, were primarily associated with cell polarity, cell adhesion, cell growth, and immune processes, which were the same biological processes that were found to differ between the *IDH*^MUT^ and *IDH*^WT^ LGGs (Supplementary Figures 3–5). Specifically, LGGs with large SVRs were found to be positively associated with a high expression of oncogenes, such as OTX1, HES1, BAG5, and the top 10 genes that were positively associated with SVR were listed in Supplementary Table 3.

### Identification of a prognostic-based radiomic signature

**Figure 3:**
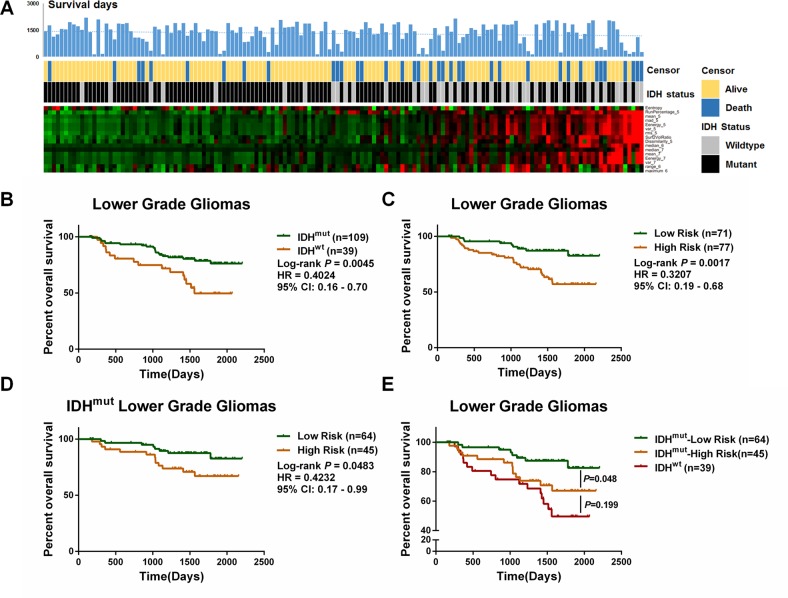
**Identification of a prognostic signature based on differential features between *IDH*^WT^ and *IDH*^MUT^ LGGs.** (**A**) The expression pattern of 16 radiomic features along with the elevation of the risk score. The corresponding survival data and *IDH* status are listed. (**B**) In 158 LGGs cohort, the *IDH*^MUT^ patients survived longer than the *IDH*^WT^ patients (*P* = 0.0045, HR = 0.4024, 95%CI:0.16–0.70). (**C**) The risk score divided the LGGs into two groups with distinct outcomes (*P* = 0.0017, HR = 0.3207, 95%CI:0.19–0.68). (**D**) *IDH*^MUT^ LGGs with a low risk score showed a favorable prognosis compared with the *IDH*^WT^ patients (*P* = 0.0483, HR = 0.4232, 95%, CI:0.17–0.99). (**E**) Further, the overall survival time of the *IDH*^MUT^ patients with a high risk score was not significantly different from that of the *IDH*^WT^ (*P* = 0.199).

Accumulating evidence has revealed that the *IDH* mutation is a crucial predictor for LGG patient outcomes. The present study also showed that the *IDH*^MUT^ LGG patients had a longer OS than the *IDH*^WT^ patients (Figure 3B). To further explore the prognostic ability of the *IDH*-specific radiomic features, we extracted a compact signature consisting of features with *P* < 0.05 after a univariate Cox regression analysis (Table 2). The β value of each significant radiomic feature was used for risk evaluation. When they had an elevated risk score, the patients were prone to have a higher mortality and a greater frequency of *IDH* mutation (Figure 3A).

**Table 2:** Fourteen prognostic radiomic features identified by Cox regression

Features	HR	β	95% CI		*P* Value
			Lower	Upper	
Mean_HLL	1.028	0.027	1.012	1.044	0.001
Median_HHL	2.977	1.091	1.548	5.726	0.001
Mean absolute deviation_HLL	1.011	0.011	1.003	1.019	0.007
RunPercentage_HLL	1.300	0.263	1.066	1.586	0.010
Range_HLH	1.001	0.001	1.000	1.002	0.013
Surface to Volume Ratio	210.673	5.352	2.872	15475.144	0.015
Entropy (group 1)	0.349	-1.052	0.149	0.817	0.015
Median_HLH	1175.998	7.070	3.749	368844.040	0.016
Energy_HLL (group 1 derived)	1.000	0.000	1.000	1.000	0.019
Mean_HHL	1.189	0.173	1.025	1.380	0.023
Variance_HLL(group 1 derived)	1.000	0.000	1.000	1.000	0.029
Dissimilarity_HLL	1.759	0.565	1.051	2.944	0.032
Root mean square_HLL	1.007	0.007	1.000	1.014	0.037
Maximum_HLH	1.001	0.001	1.000	1.003	0.047

HR = Hazard Ratio; 95% CI = 95% Confidence Interval.

Using the calculated risk score, we divided the LGG patients into high-risk and low-risk groups. Patients with a high-risk score had a worse prognosis (Figure 3C, HR = 0.3207, *P* = 0.0017). Intriguingly, *IDH*^MUT^ patients could be further categorized into *IDH*^MUT^-high risk and *IDH*^MUT^-low risk groups with significantly different OSs (Figure 3D, HR = 0.4232, *P* = 0.0483). A multivariate Cox analysis also demonstrated that the radiomic risk score could serve as a prognostic indicator for LGGs (Table 3). Moreover, the OS was not significantly different between the *IDH*^WT^–high risk groups and the *IDH*^MUT^-high risk groups (Figure 3E, *P* = 0.199), further emphasizing the prognostic value of the *IDH*-specific radiomic signature.

**Table 3:** Univariate and multivariate Cox analysis in Lower Grade Glioma

Variables	Univariate Cox Regression			Multivariate Cox Regression		
	HR	95% CI	P value	HR	95% CI	P value
Sex						
Female vs. Male	0.722	0.363-1.439	0.355	0.870	0.428-1.767	0.699
Age	0.978	0.944-1.012	0.202	0.991	0.959-1.024	0.567
WHO Grade						
II vs. III	0.337	0.177-0.643	0.001	0.420	0.213-0.827	0.012
IDH status						
MUT vs. WT	0.402	0.209-0.770	0.006	0.538	0.269-1.078	0.081
Risk Score						
Low vs. High	0.806	0.727-0.894	<0.001	0.875	0.780-0.981	0.022

**Figure 4:**
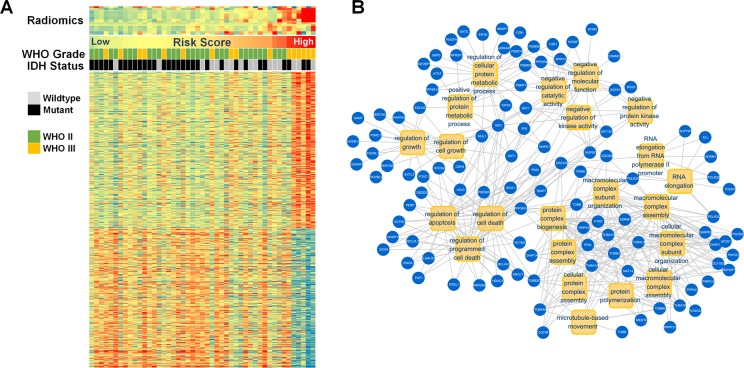
**Gene annotation of 48 patients with radiomic and transcriptome data.** (**A**) The radiomic features, clinical characteristics, and associated genes are presented. (**B**) The positively associated genes (blue) that participated in GO in terms (yellow) of apoptosis, cell growth, and metabolic processes.

**Figure 5:**
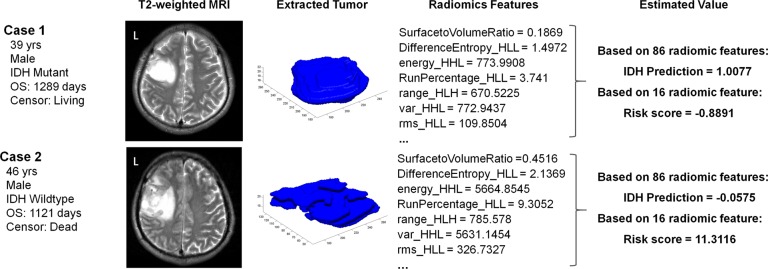
**Case examples of LGG patients with T2-weighted images. Case 1 was a 39-year-old male with an *IDH* mutant LGG.** This patient was classified into the *IDH*^MUT^ group with a relatively low risk score based on the radiomic features. In contrast, case 2 was a 46-year-old male with an *IDH* wildtype LGG, who was correctly classified into the *IDH*^WT^ group with a high risk score.

A radiogenomic analysis found that a high-risk score was positively associated with genes that included *ERCC1*,* G6PD*,* SOX9, and EGLN2,* which are primarily enriched during the regulation of programmed cell death, cell growth, and metabolic processes. This could partially account for the radiological malignancy of the high-risk group (Figure 4). Representative samples of T2-weighted images with relevant radiomics and clinical features are presented in Figure 5. The first case was a 39-year old male patient with IDH mutant LGG. This patient was classified into the *IDH*^MUT^ group with a relatively low radiomics risk score. Case 2 was a 46-year-old male with the *IDH* wildtype LGG who was correctly classified into the *IDH*^WT^ group with a high-risk score.

Additionally, the current study revealed that the radiomic risk score was significantly positively correlated with glioma stem cell markers such as TWIST1 (R = 0.503, P < 0.001) and CD133 (R = 0.346, P = 0.016) (Supplementary Figure 6).

## DISCUSSION

By assessing the comprehensive characteristics of the entire tumor noninvasively, MR imaging is currently an indispensable approach for glioma diagnosis and treatment monitoring. The development of computational methodologies has successfully converted routine MR images to informative descriptors, substituting a quantitative and objective modality for traditionally qualitative and subjective methods. In the present study, we analyzed 431 T2-weighted radiomic features in 158 LGG patients and identified an *IDH*-specific radiomic signature. An integrated analysis of both radiomic and transcriptomic data indicated that these radiomic features could reflect the tumor immune response, adhesion, and several malignant biological processes, all of which are in accord with behaviors that differentiate between *IDH*^MUT^ and *IDH*^WT^ LGGs. Furthermore, these *IDH*-specific radiomic features could be utilized to establish a prognostic evaluation model. The *IDH*^MUT^ patients with a low risk score showed a significantly longer OS than the *IDH*^MUT^ patients with a high-risk score.

Medical imaging holds great promise for monitoring the progression of disease and the therapeutic response because it can noninvasively provide a more comprehensive view of tumors and can be performed repeatedly in routine practice [26]. However, unlike our quantitative radiomics analyses, the conventional evaluation of MR images is subjectively based on the experience of the radiologists and neurosurgeons, leading to a lack of conformity between different clinical centers. In this study, we identified the quantitative radiomics features that were differentially expressed in two *IDH* phenotypes. The consistency of the findings was validated by an independent cohort of 102 LGGs patients. Intriguingly, some of the differential radiomic features could also be observed in an in vivo glioma model. Several reasons may contribute to this phenomenon. First, IDH1 mutation results in dramatically elevated levels of 2HG, a potential oncometabolite, which could influence the whole metabolic profile.[34] Secondly, IDH1 mutation is sufficient to establish the glioma hypermethylator phenotype, which is a powerful determinant of tumor pathogenicity [35].

We utilized the integrative analysis of radiomic and transcriptomic data to decipher radiological characteristics that could be associated with biological processes and gene expression. The SVR, also called the surface-area-to-volume ratio, is the amount of surface area per unit volume of an object. For a given volume, the object with the smallest surface area (namely, with the smallest SVR) is a sphere. In contrast, objects with tiny spikes have a very large surface area for a given volume. In keeping with its biological significance, LGGs with large SVRs were found to be positively associated with a high expression of oncogenes, such as *OTX1*, *HES1*, *BAG5*; these genes are involved in biological processes that include the immune process and responses to stimuli. Similarly, a previous study revealed that patients with spherical tumors survived significantly longer than those with irregular tumor surface in glioblastomas, which indicated that tumors with irregular surface could be more malignant than spherical tumors [36]. Another radiomic parameter enriched in the *IDH*^WT^ LGGs was Entropy, which describes irregularity of pixel values within the tumor area (higher values suggesting increasing irregularity). Entropy is a promising quantitative imaging biomarker for capturing cancer imaging phenotype, and a higher Entropy was found to be associated with higher tumor metabolism, higher tumor grade, worse prognosis, and worse treatment response [37–39]. The current study revealed that Entropy was positively associated with transcription, chromatin organization and other biological functions (Supplementary Figure 3). The current finding is in accordance with previous studies.

The discovery that *IDH* mutations lead to accumulation of the oncometabolite 2-HG indicates an oncogenic role of the *IDH* mutation in the genesis of malignant brain tumors [34]. Therefore, the biological functions of the *IDH* mutation have attracted attention worldwide. Researchers suggested that the *IDH* mutation is correlated with the regulation of HIF1 [40], the escape immune system [41], and aggregation [42]. In the present study, RNA sequencing data from both TCGA and CGGA revealed that *IDH*^MUT^ and *IDH*^WT^ LGGs showed differences in immune response, vascular development, adhesion, and even proliferation, which further confirmed the genotypic and phenotypic differences between the two groups. Importantly, these genetic alterations and biological behaviors may be manifested radiologically, supporting a preoperative and noninvasive strategy for *IDH* prediction.

A recent study showed that *IDH* mutant high grade gliomas were more amenable to a complete resection of enhancing tumors and had an improved survival with the resection of non-enhanced tissues [43]. Therefore, preoperatively evaluating the *IDH* status may be beneficial for surgical decision making and for developing selective targeted therapy [25]. An increasing number of studies indicate that patients harboring *IDH* mutations have a better prognosis. However, some *IDH*^MUT^ patients had a shorter survival than some *IDH*^WT^ patients. In the present work, we used radiomic features to promote the prognostic prediction. The results implied that a combination of genetic alterations and radiomic changes could potentially provide a non-invasive methodology for genotype detection. What is more, the worse prognosis of high risk patients could be partially attributable to cell growth, metabolic processes, and programmed cell death, providing a new approach for developing therapeutic targets.

There are several limitations in the present study. We used T2-weighted MR images, a routine type of imaging for clinical glioma management, for the radiomic analysis. Although we normalized the radiological data, weighting the imaging data may not reflect the actual situation in the brain tissue, and the data can and does vary between MR scanners. A quantitative approach, such as T2 mapping, would be more suitable for the analysis of the MR images. Moreover, multi-dimensional data (copy number variance, methylation, proteomic profile, etc.) and a larger cohort would help to further delineate the radiomic and genomic landscape of glioma, and multi-regional samplings would help to obtain more precise results. Additionally, the specific radiomic features of tumors in various pathological types is an interesting topic to be investigated in future studies.

## CONCLUSIONS

In conclusion, we demonstrated that radiomic features could serve as an alternative approach for *IDH* phenotype classification in LGG patients. The MR imaging is a routine examination for gliomas, and quantitative radiomic and radiogenomic analyses can potentially provide a noninvasive modality for prognosis prediction and phenotypic monitoring. As the whole process of radiomic analysis takes less than 10 minutes for an individual case and can be practice automatically with out further cost, this newly developed technique is increasingly applied to assisting clinical diagnosis and decision making.

## MATERIALS AND METHODS

### Patients and samples

**Figure 6:**
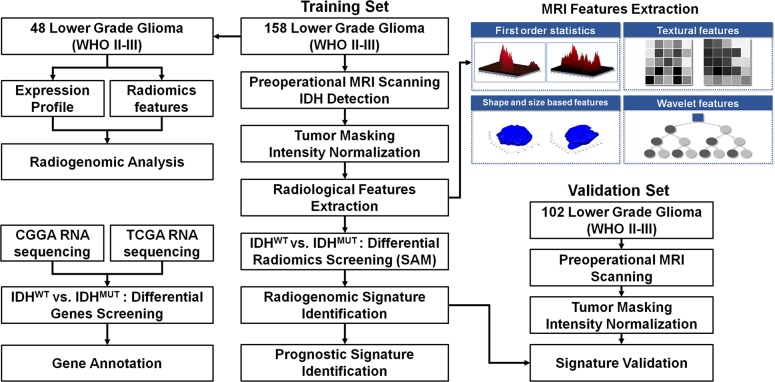
The workflow of the radiogenomic analysis for the identification and validation of the *IDH* mutation-specific radiomic signature in lower grade gliomas.

A total of 158 LGG patients who underwent surgical treatment between March 2006 and December 2012 were retrospectively selected as a training set. An additional 102 LGG patients who underwent surgical treatment from January 2016 to June 2016 were prospectively included as a validation set. The inclusion criteria were as follows: (1) pathologically confirmed lower grade glioma; (2) available IDH status; (3) available high-resolution preoperative T2-weighted MR images; (4) available clinical characteristics. The basic clinical characteristics of the patients, including age, gender, WHO grade, and tumor location are summarized in Table 1. The study was approved by the institutional review board of Beijing Tiantan Hospital. The design of the present study is illustrated in Figure 6.

### Tumor masking and normalization

The tumor masking was conducted as previously described [44]. Most of the MR images were obtained using a Trio 3.0T scanner (Siemens, Erlangen, Germany), and the remaining clinical structural images were acquired on a Magnetom Verio 3T (Siemens AG, Erlangen, Germany). The T2-weighted image parameters were as follows: repetition time = 5800 ms; echo time = 110 ms; flip angle = 150 degrees; 24 slices; field of view = 240×188 mm^2^; voxel size = 0.6×0.6×5.0 mm^3^; matrix = 384×300. Tumors were traced directly using MRIcron (http://www.mccauslandcenter.sc.edu/mricro/mricron). Masks of the brain tumors were drawn on each patient’s T2-weighted images in native space by two board-certified neuroradiologists (K.W. and X.C.), who were blind to the patients’ clinical information. Areas that produced abnormal hyperintense signals on the T2-weighted images were identified as LGG tumor areas. When a greater than 5% discrepancy existed between these two masks, the masks utilized were determined by a senior neuroradiologist (S.L.). The intensities of the voxels in each tumor mask were normalized to the z distribution ([gray value – mean gray value] / SD), on the individual level, to ensure consistency in the distribution of the gray values among the cases in the cohort.

### Quantitative radiological features extraction and selection

The extraction of the radiomic features was performed as previously reported [26]. A total of 431 image features were extracted from the tumor masks. The features were categorized into four groups. Group 1 quantitatively described the distribution of voxel intensities in the MR image using 14 descriptors. Group 2 described 8 three-dimensional features based on the shape of the tumor regions. Group 3 described textual features for quantifying intra-tumor heterogeneity, which were calculated from gray level co-occurrence (GLCM, 22 descriptors) and gray level run-length (GLRLM, 11 descriptors) texture matrices. Group 4 calculated the intensity and textural features from wavelet decompositions of the original image. All the algorithms were implemented in MATLAB (2014a).

### Transcriptomic comparison between IDH^WT^ and IDH^MUT^ LGG

All available whole genome mRNA sequencing data of LGG patients and clinical information were acquired from the Chinese Glioma Genome Atlas database (http://www.cgga.org.cn) and the Cancer Genome Atlas database (http://cancergenome.nih.gov/). The differentially expressed genes were selected by a significance analysis of the microarray (SAM) algorithm using R programming language (http://cran.r-project.org), and with the criteria of fold change > 20% and false discovery rate (FDR) < 0.05 (Benjamini-Hochberg). Finally, the genes significantly overexpressed in the *IDH*^WT^ group (CGGA, 1509; TCGA, 1262) or in the *IDH*^MUT^ group (CGGA, 914; TCGA, 582) were processed using Gene Cluster and Gene Treeview software to construct a heatmap, and the online Database for Annotation, Visualization, and Integrated Discovery (DAVID, http://david.ncifcrf.gov/) program [45] for the gene ontology (GO).

### Xenograft model of glioma and radiomic analysis

Lentiviral vectors carrying *IDH1* wildtype or *IDH1* R132H mutant cDNA sequences were transduced into the U87MG cells with polybrene (Sigma), as previously described [42]. Stably transduced cells were selected during three days of puromycin (Sigma) treatment. The Western Blot was conducted to verify the expression of the mutant *IDH1* enzyme using a cell lysate and the *IDH1*^R132H^ antibody (1:200, DIA-H05, Dianova). Then, *IDH1* wild type or R132H mutant U87MG cells (5 × 10^5^ cells per mouse in 5 µL) were intracranially injected into 5 to 6 weeks old female nude mice (Beijing Vital River Laboratory Animal Technology), as described earlier [46]. After 40 days, the tumors were measured using a 7T MR Image System (Bioclinscan, Bruker). The relevant T2-weighted MR images were subjected to radiomic feature extraction and analysis. After tumor segmentation, a total of 431 radiomic feature were extracted from the T2-weighted MR images from each mouse. The differentially expressed radiomic features were selected using SAM algorithm, with the criteria of false discovery rate (FDR) < 0.05 (Benjamini-Hochberg).

### IDH phenotype classification

First, similar to the radiomic analysis in xenograft model, SAM algorithm was conducted on the 431 radiomic features to select the differentially expressed radiomic features between IDH^WT^ and IDH^MUT^ tumors, with the criteria of false discovery rate (FDR) < 0.05 (Benjamini-Hochberg). Next, the selected differentially expressed radiomic features were utilized for IDH phenotype classification. The classification process was conducted using a logistic regression model (Y = expr _feature 1_ × β_ feature 1_ + expr _feature 2_ × β _feature 2_ + … + expr_ feature n_ × β_ feature n_ + ε) based on the MATLAB (2014a) software. In this model, ‘Y’ represents the IDH status (1 indicates mutation, 0 indicates wildtype), while ‘expr’ represents the expression value of each radiomic feature. β represents the model parameter to be estimated, and ε is the estimated residual. The prediction results were further interpreted using the receiver operating characteristic (ROC) curve. To select the most predictive parameters, the logistic regression algorithm was applied repeatedly. Using the dimensionality reduction principle, the radiomic feature with the highest P value in predicting IDH status was excluded from the model each time until features with the best predictive effect were identified. The signature derived from the training set was subsequently applied to the validation set.

### Identification of the prognosis-based signature

The prognostic values for each *IDH*-specific feature in patients with LGG were calculated using a univariate Cox regression after elimination of the patients with an OS of less than 30 days. The significant radiomic features with P values < 0.05 were selected to develop a prognosis-based signature. The risk score was calculated using the linear combination of the selected features weighted by the regression coefficient derived from the univariate Cox regression analysis (β), referring to previous studies [47, 48]. The risk score for the OS of each individual was calculated as follows: Risk score = expr _feature 1_ × β _feature 1_ + expr _feature 2_ × β _feature 2_ + … + expr _feature n_ × β _feature n_. ‘Expr’ represents the expression value of each radiomic feature. The ‘expr’ and β used in methods 2.7 have no relationship with those used in methods 2.6.

We next divided the patients into high-risk and low-risk groups based on the risk score. The cutoff value was determined when the P value was the smallest in log-rank test in the training set, and then the cut-off value was fixed and applied to all Kaplan-Meier curve analyses.

### Radiogenomic analysis

Forty-eight LGG patients involved in the radiomic analysis were subjected to an Agilent Whole Human Genome Array analysis according to the manufacturer’s instructions [49]. The data were acquired using the Agilent G2565BA Microarray Scanner System and Agilent Feature Extraction Software (version 9.1). The probe intensities were normalized using GeneSpring GX 11.0. The IDH mutations of the training data set were assessed by pyrosequencing, while the IDH status for the validation data set was identified by immunohistochemistry (IDH1^R132H^, DIA-H05, Dianova).

Radiogenomic analysis was further performed. The Pearson correlation coefficients were calculated between the genes and IDH-associated radiomic features. The association was identified to be statistically significant when the absolute value of Pearson correlation coefficient was > 0.4 and the P value was < 0.05. Gene ontology (GO) analysis was conducted to investigate the underlying biological processes of the radiomic features based on the DAVID Bioinformatics Resources (http://david.ncifcrf.gov/). The top 200 positive/negative genes that were significantly associated with each feature were subjected to GO analysis to reveal the underlying biological processes of each feature. Using this method, the underlying biological processes of the risk score and the differentially expressed features in xenograft model were also investigated. In addition, the relationship between the radiomic risk score and glioma stem cell signatures was also assessed with using the Pearson correlation analysis.

### Statistics

The significant differences between the two groups were estimated using a Student’s *t*-test. Chi-square and Fisher’s exact tests were used to compare the frequencies between the groups. The OS curves were plotted according to the Kaplan–Meier method, with the log-rank test applied for comparison. A Cox regression was used to determine the prognostic value of each radiomic feature for the OS in LGG patients. All the differences were considered statistically significant at the two-sided *P* < 0.05 level.

## SUPPLEMENTARY MATERIALS

Supplementary Figure 1

Supplementary Figure 2

Supplementary Figure 3

Supplementary Figure 4

Supplementary Figure 5

Supplementary Figure 6

Supplementary Table 1

Supplementary Table 2

Supplementary Table 3
