# Supplementary material for: IDH mutation-specific radiomic signature in lower-grade gliomas
Source: Aging (Albany NY). 2019 Jan 29;11(2):673–96. doi: 10.18632/aging.101769 (PMC6366985; doi:10.18632/aging.101769)
Supplement: Supplementary Figure 1 [file aging-11-101769-s001.pdf]

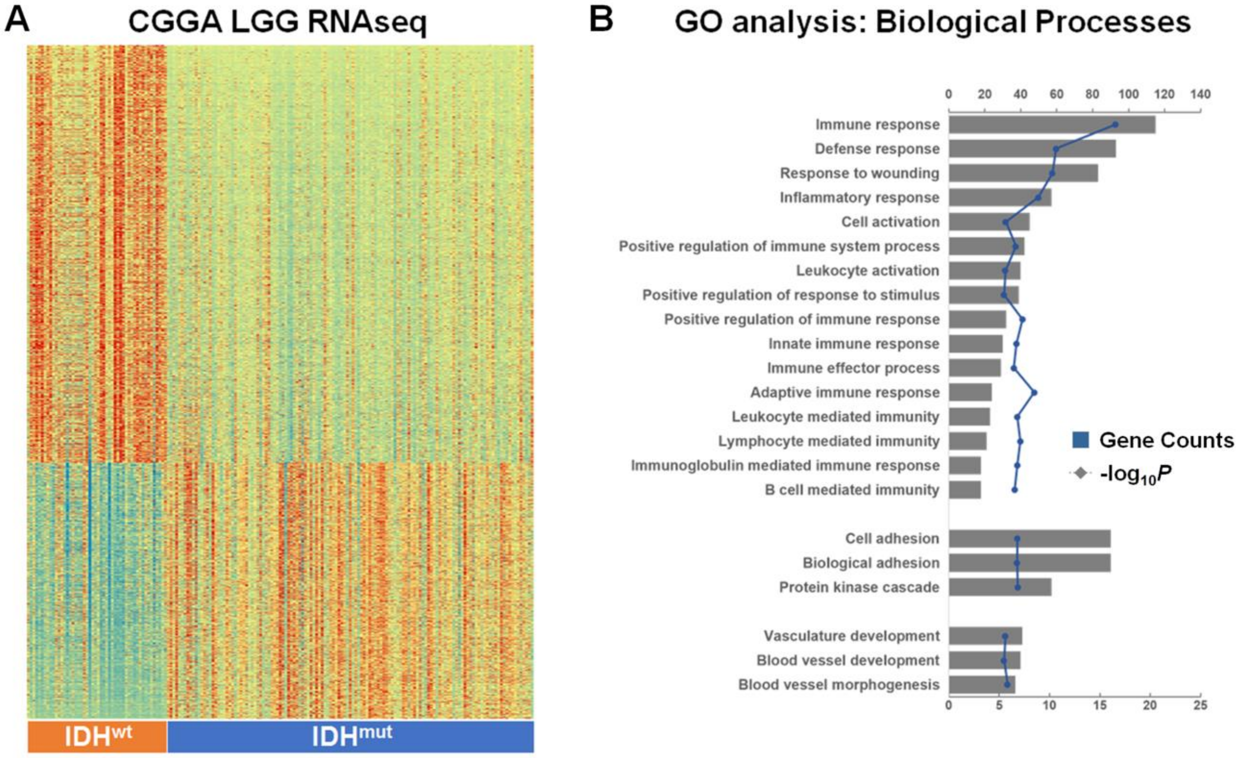

Supplementary Figure 1. The transcriptomic distinction between *IDH*^{MUT} and *IDH*^{WT} LGGs in the CGGA cohort. (**A**) Differentially expressed genes determined by the Pearson correlation algorithm. (**B**) Highly expressed genes in the *IDH*^{WT} LGGs were investigated by gene annotation. The enriched biological processes included the immune response, cell adhesion, and vascular related terms.
