# Supplementary material for: IDH mutation-specific radiomic signature in lower-grade gliomas
Source: Aging (Albany NY). 2019 Jan 29;11(2):673–96. doi: 10.18632/aging.101769 (PMC6366985; doi:10.18632/aging.101769)
Supplement: Supplementary Figure 2 [file aging-11-101769-s002.pdf]

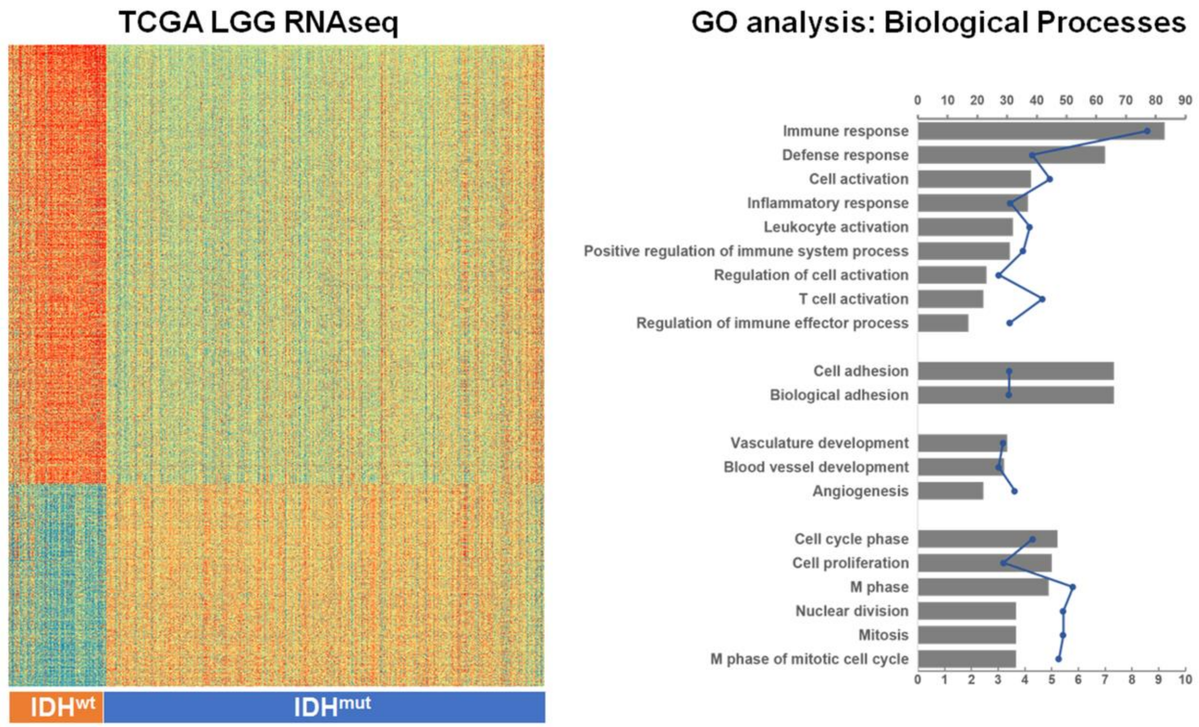

Supplementary Figure 2. The transcriptomic distinction between the *IDH*^{MUT} and *IDH*^{WT} LGGs in the TCGA cohort.
