# Supplementary material for: IDH mutation-specific radiomic signature in lower-grade gliomas
Source: Aging (Albany NY). 2019 Jan 29;11(2):673–96. doi: 10.18632/aging.101769 (PMC6366985; doi:10.18632/aging.101769)
Supplement: Supplementary Figure 4 [file aging-11-101769-s004.pdf]

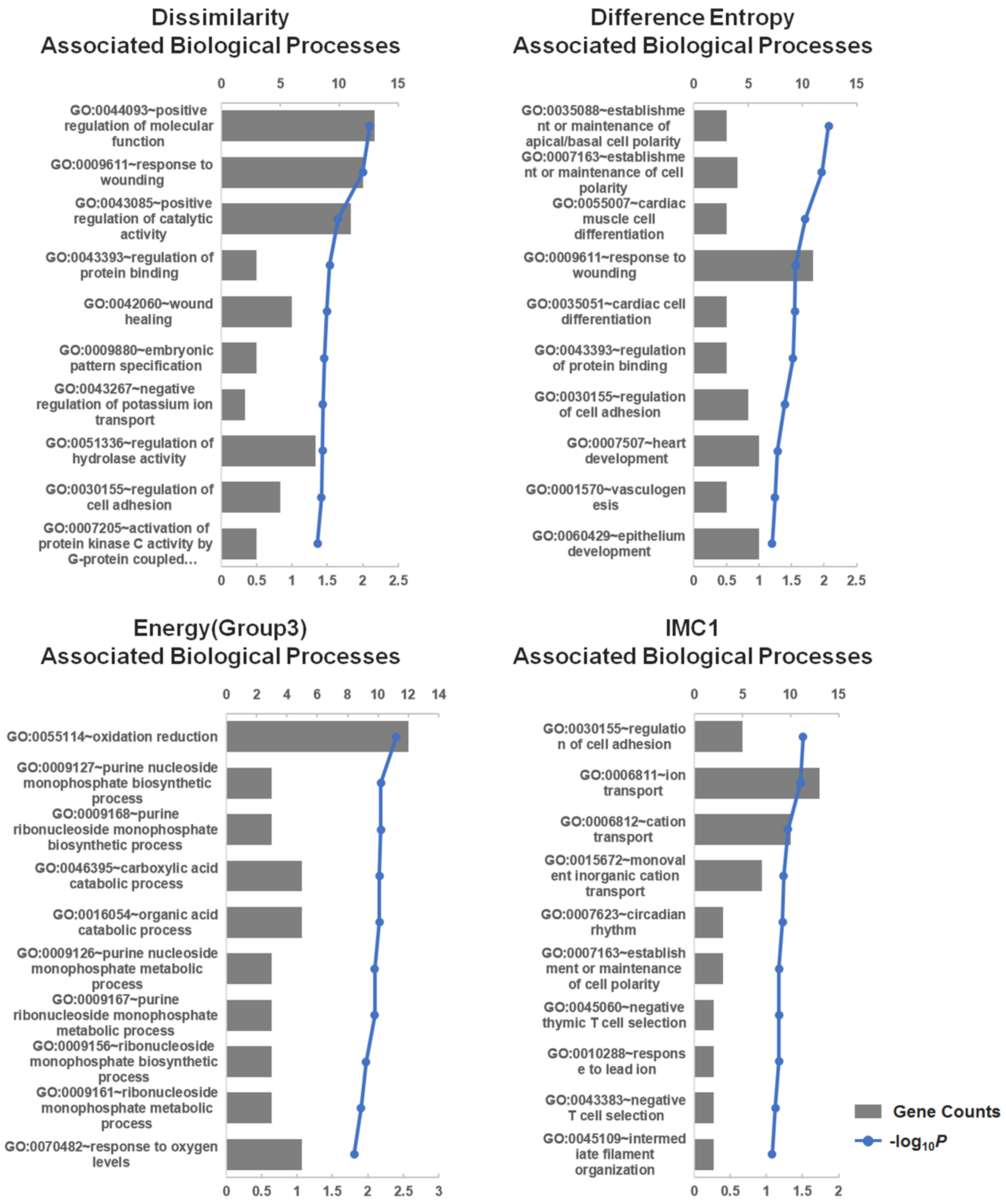

Supplementary Figure 4. The associated genes and relevant GO result of Group 3 descriptors: Dissimilarity, Difference Entropy, Energy, and IMC1 (Informational measure of correlation 1).
