# Supplementary material for: IDH mutation-specific radiomic signature in lower-grade gliomas
Source: Aging (Albany NY). 2019 Jan 29;11(2):673–96. doi: 10.18632/aging.101769 (PMC6366985; doi:10.18632/aging.101769)
Supplement: Supplementary Figure 6 [file aging-11-101769-s006.pdf]

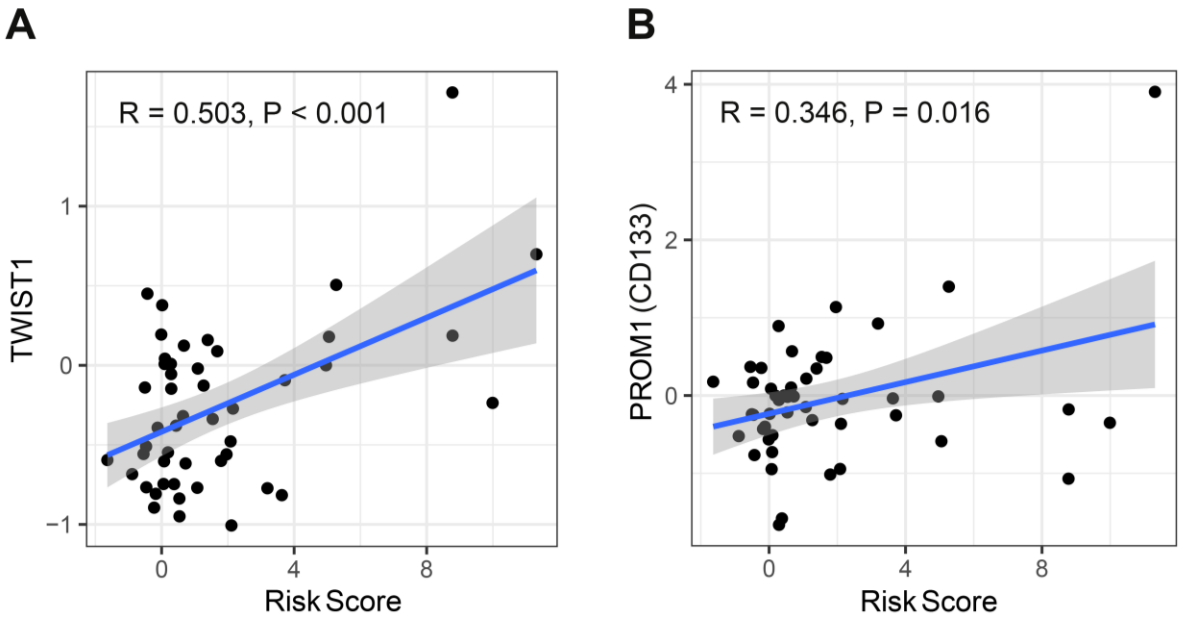

Supplementary Figure 6. Pearson correlation analysis was performed between the radiomics risk score and the glioma stem cell markers TWIST1 and CD133.
