# Supplementary material for: IDH mutation-specific radiomic signature in lower-grade gliomas
Source: Aging (Albany NY). 2019 Jan 29;11(2):673–96. doi: 10.18632/aging.101769 (PMC6366985; doi:10.18632/aging.101769)
Supplement: Supplementary Table 1 [file aging-11-101769-s007.pdf]

Supplementary Table 1. Differential Radiomics features (n=146) between IDH mutant and IDH wildtype groups in the training set.

Features Type			
Shape and size based features			
Surface to Volume Ratio			
First order statistics			
Energy	Entropy	Median	
Mean	Root mean square		
Gray Level Co-occurrence Matrix			
Contrast	Dissimilarity		
Energy	Entropy		
Difference entropy	IMC1		
Gray Level Run-Length Matrix			
Short run emphasis	Run Percentage		
Wavelet features			
Cluster shade_HLL	Energy_HHL (group 1 derived)	Maximum_LLH	Root mean square_HLL
Contrast_LLL	Energy_HHL (group 3 derived)	Maximum_LHL	Root mean square_HLH
Contrast_LLH	Energy_HHH (group 1 derived)	Maximum_LHH	Root mean square_HHL
Contrast_LHL	Energy_HHH (group 3 derived)	Maximum_HLL	Root mean square_HHH
Contrast_HLH	Entropy_LLL (group 1 derived)	Maximum_HLH	Run percentage_LLL
Contrast_HHL	Entropy_LLL (group 3 derived)	Maximum_HHL	Run percentage_LHL
Contrast_HHH	Entropy_LLH (group 1 derived)	Maximum_HHH	Run percentage_LHH
Difference entropy_LLL	Entropy_LLH (group 3 derived)	Mean_LLL	Run percentage_HLL
Difference entropy_LLH	Entropy_LHL (group 1 derived)	Mean_LLH	Run percentage_HLH
Difference entropy_LHL	Entropy_LHL (group 3 derived)	Mean_LHL	Run percentage_HHH
Difference entropy_HLL	Entropy_LHH (group 1 derived)	Mean_LHH	Short run emphasis_LLL
Difference entropy_HLH	Entropy_LHH (group 3 derived)	Mean_HLL	Short run emphasis_LLH
Difference entropy_HHL	Entropy_HLL (group 1 derived)	Mean_HLH	Short run emphasis_LHL
Difference entropy_HHH	Entropy_HLL (group 3 derived)	Mean_HHL	Short run emphasis_LHH
Dissimilarity_LLL	Entropy_HLH (group 1 derived)	Mean_HHH	Short run emphasis_HLL
Dissimilarity_LLH	Entropy_HLH (group 3 derived)	Median_LLL	Short run emphasis_HLH
Dissimilarity_LHL	Entropy_HHL (group 1 derived)	Median_LLH	Short run emphasis_HHL
Dissimilarity_HLL	Entropy_HHL (group 3 derived)	Median_LHL	Short run emphasis_HHH
Dissimilarity_HLH	Entropy_HHH (group 1	Median_LHH	Standard deviation_LLH

Features Type			
	derived)		
Dissimilarity_HHL	Entropy_HHH (group 3 derived)	Median_HLH	Standard deviation_LHL
Dissimilarity_HHH	IMC1_LLL	Median_HHL	Standard deviation_LHH
Energy_LLL (group 1 derived)	IMC1_HLL	Median_HHH	Standard deviation_HLL
Energy_LLL (group 3 derived)	IMC1_HLH	Minimum_LLL	Standard deviation_HLH
Energy_LLH (group 1 derived)	IMC1_HHL	Range_LLH	Standard deviation_HHL
Energy_LLH (group 3 derived)	IMC1_HHH	Range_LHL	Standard deviation_HHH
Energy_LHL (group 1 derived)	Mean absolute deviation_LLH	Range_HLL	Sum entropy_HHL
Energy_LHL (group 3 derived)	Mean absolute deviation_LHL	Range_HLH	Variance_LLH (group 1 derived)
Energy_LHH (group 1 derived)	Mean absolute deviation_LHH	Range_HHL	Variance_LHL (group 1 derived)
Energy_LHH (group 3 derived)	Mean absolute deviation_HLL	Range_HHH	Variance_LHH (group 1 derived)
Energy_HLL (group 1 derived)	Mean absolute deviation_HLH	Root mean square_LLL	Variance_HLL (group 1 derived)
Energy_HLL (group 3 derived)	Mean absolute deviation_HHL	Root mean square_LLH	Variance_HLH (group 1 derived)
Energy_HLH (group 1 derived)	Mean absolute deviation_HHH	Root mean square_LHL	Variance_HHL (group 1 derived)
Energy_HLH (group 3 derived)	Maximum_LLL	Root mean square_LHH	Variance_HHH (group 1 derived)

IMC1 = Informational measure of correlation 1.
