# Supplementary material for: IDH mutation-specific radiomic signature in lower-grade gliomas
Source: Aging (Albany NY). 2019 Jan 29;11(2):673–96. doi: 10.18632/aging.101769 (PMC6366985; doi:10.18632/aging.101769)
Supplement: Supplementary Table 2 [file aging-11-101769-s008.pdf]

Supplementary Table 2. Forty differentially expressed radiomic features between IDH mutant and IDH wildtype groups in the xenograft model.

Number	Features
1	Energy (Group 1)
2	Energy_LLL (Group 1 derived)
3	Energy_LHL (Group 1 derived)
4	Energy_HLH (Group 1 derived)
5	Energy_HHL (Group 1 derived)
6	Energy_HHH (Group 1 derived)
7	Mean absolute deviation_HHL
8	Mean absolute deviation_HHH
9	Maximum_LLL
10	Maximum_LHL
11	Maximum_LHH
12	Maximum_HLL
13	Maximum_HLH
14	Maximum_HHL
15	Maximum_HHH
16	Mean
17	Mean_LLL
18	Mean_HHL
19	Median
20	Median_LLL
21	Range_LLH
22	Range_LHL
23	Range_HLL
24	Range_HLH
25	Range_HHL
26	Range_HHH
27	Root mean square
28	Root mean square_LLL
29	Root mean square_LHL
30	Root mean square_HLH
31	Root mean square_HHL
32	Root mean square_HHH
33	Standard deviation_LHL
34	Standard deviation_HLH
35	Standard deviation_HHL
36	Standard deviation_HHH
37	Variance_LHL (Group 1 derived)
38	Variance_HLH (Group 1 derived)
39	Variance_HHL (Group 1 derived)
40	Variance_HHH (Group 1 derived)
