# Supplementary material for: IDH mutation-specific radiomic signature in lower-grade gliomas
Source: Aging (Albany NY). 2019 Jan 29;11(2):673–96. doi: 10.18632/aging.101769 (PMC6366985; doi:10.18632/aging.101769)
Supplement: Supplementary Table 3 [file aging-11-101769-s009.pdf]

Supplementary Table 3. Top 10 genes that were positively associated with surface to volume ratio.

Genes	Pearson correlation coefficients	*P*
OTX1	0.548373009	5.46E-05
SP8	0.535831595	8.68E-05
BAG5	0.528369318	0.000113355
C9orf144B	0.507442171	0.000232145
GPR37L1	0.501972823	0.000277878
RAB4B	0.489037137	0.000420165
AHCYL2	0.48877379	0.000423645
KIAA0090	0.483516067	0.000498797
ATP1A4	0.475963747	0.000627815
